# Can Compression Garments Reduce Inter-Limb Balance Asymmetries?

**DOI:** 10.3389/fnhum.2022.835784

**Published:** 2022-02-17

**Authors:** Frédéric Noé, Kévin Baige, Thierry Paillard

**Affiliations:** Université de Pau et des Pays de l’Adour, E2S UPPA, MEPS, Tarbes, France

**Keywords:** laterality, symmetry, posture, balance control, compression garments

## Abstract

Sensory cues provided by compression garments (CG) can improve movement accuracy and potentially reduce inter-limb balance asymmetries and the associated risk of injury. The aim of this study was to analyze the effects of CG wearing on inter-limb balance asymmetries. The hypothesis was that CG would reduce inter-limb balance asymmetries, especially in subjects with high level of asymmetries. Twenty-five sportsmen were recruited. They had to stand as motionless as possible in a one-leg stance in two postural tasks (stable and unstable), while wearing CG or not. Asymmetry indexes were calculated from center of foot pressure parameters. The effects of CG wearing were analyzed according to participants’ baseline level of asymmetry (i.e., without wearing CG) with correlation analyses. A qualitative analysis was also performed after a dichotomization procedure to check for a specific influence of CG on the dominant and non-dominant leg. Inter-limb balance asymmetries were reduced with CG in participants with high levels of asymmetries at baseline. However, asymmetries were increased with CG in participants with low levels of asymmetries at baseline. The dominant leg was more affected by this negative effect. CG wearing could reduce inter-limb balance asymmetries and the related injury risk in subjects with high levels of inter-limb balance asymmetries at baseline. Nevertheless, CG should not be used in individuals with low baseline balance asymmetries since it can increase asymmetries in these subjects, likely by confusing and overloading the sensorimotor processing on the dominant leg.

## Introduction

Asymmetries between two limbs are prevalent in human movement. They are characterized by a difference in performance between the right and left limbs and/or the preferential use of one side of the body (i.e., left or right) to perform a motor action (Schneiders et al., 2010; Bishop et al., 2018). The etiologies of inter-limb asymmetries are related to lateralization in sensorimotor control originating from cerebral hemispheric asymmetry (Promsri et al., 2018, 2019; Marcori et al., 2020; Liu et al., 2021) and biomechanical factors (e.g., bilateral asymmetry in the bones length, imbalance of muscle strength between the left and right limb) (Auerbach and Ruff, 2006; Bishop et al., 2018). Inter-limb asymmetries can be modulated by numerous factors. Injuries and pathologies such as anterior cruciate ligament injuries (Mohammadi et al., 2012) or scoliosis (Yang et al., 2013) increase inter-limb asymmetries. Subjects affected by neurologic conditions (e.g., stroke and multiple sclerosis) and individuals with experience in asymmetric sports (e.g., soccer, volley-ball, and hand-ball) also demonstrate pronounced inter-limb asymmetries (Chisholm et al., 2011; Paillard and Noé, 2020; Pau et al., 2021).

Inter-limb asymmetries can have a negative impact on sports performance and are associated with a high incidence of lower-limb injury, especially if asymmetries concern balance control (Stiffler et al., 2017; Bishop et al., 2018; Promsri et al., 2019). Actually, inter-limb balance asymmetries may impair the ability to shift the body weight onto one leg thus negatively impacting sports-related actions such as sidestepping or changes of direction and predisposing sportspeople to non-contact lower-limb injury (Bishop et al., 2018; Dos’Santos et al., 2019; Promsri et al., 2019). Therefore, training strategies such as balance training, resistance training or warm-up programs, can be implemented to minimize inter-limb balance asymmetries, improve balance control and reduce the relative risk of sports-related non-contact injury (Bishop et al., 2018; Pardos-Mainer et al., 2019; Madruga-Parera et al., 2020). Affordable external devices that interact with cutaneous receptors such as compression garments (CG) can also acutely improve balance control (Kuster et al., 1999; Michael et al., 2014; Woo et al., 2017; Baige et al., 2020). The constriction provided by CG acts as a mechanically supportive framework that can activate interacting cutaneous mechanoreceptors that individually would not have been activated (Baige et al., 2020), thus improving movement accuracy during tasks that include a large somatosensory component (Hasan et al., 2016; Ghai et al., 2018; Broatch et al., 2021) and offering a potential benefit in reducing inter-limb balance asymmetries. Nevertheless, to our knowledge, no study has specifically investigated the effects of CG wearing on inter-limb balance asymmetries.

Hence, this study was undertaken to investigate the effects of CG on inter-limb balance asymmetries in individuals with extensive experience in an asymmetric sport such as handball players. Handball is a sport involving asymmetric motor actions (lower and upper limbs) that can accentuate inter-limb asymmetries, which might negatively affect athletic performance and increase the risk of lower extremity injuries (Steib et al., 2016; Paillard, 2017; Barrera-Domínguez et al., 2021). Thus, a passive intervention such as CG wearing is hypothesized to reduce inter-limb balance asymmetries, especially in subjects with a high level of asymmetry, and could represent a cost effective sport-related injury prevention strategy.

## Materials and Methods

### Participants

Twenty-five young handball players (age: 27.1 ± 6.9 years old, height: 184.8 ± 8.1 cm; body mass: 87.2 ± 15.3 kg; mean ± SD) who reported no neuromuscular problems in the past 2 years volunteered for the current study. Participants were recruited from two regional semi-professional teams. They were asked to avoid strenuous activity and the ingestion of alcohol or/and excitatory substances 24 h before the experimental session. A written informed consent was obtained from all participants before starting the experiment, which was in accordance with the Declaration of Helsinki. All procedures were approved by and performed in accordance with the relevant guidelines and regulations of the University of Pau and Pays de l’Adour Ethics Committee.

### Apparatus and Procedure

Participants were asked to sway and move as little as possible when standing barefoot in a one-leg stance for 25 s on a force platform (Stabilotest^®^ Techno Concept™, Mane, France) which sampled the displacements of the center of foot pressure (position of the point of application of the vertical ground reaction force) at 40 Hz. Two postural tasks were conducted: a stable task where participants stood directly on the force platform (on stable ground) with their eyes closed (while keeping their gaze straight ahead) and an unstable task where they stood with their eyes open (while looking at a fixed level target at a distance of 2 m) on a wobble board with a diameter of 40 cm and a height of 8 cm (Balance-board, Sissel^®^ GmbH, Bad Dürkheim, Germany) which was placed on the force platform to generate instability. For accurate and similar feet positioning between all participants, the platform and the wobble board were marked with a central horizontal line, and participants were required to align the middle of the foot with the mark (the middle of each foot was delimited beforehand). The center of the wobble board was also aligned with the center of the force platform.

**FIGURE 1:**
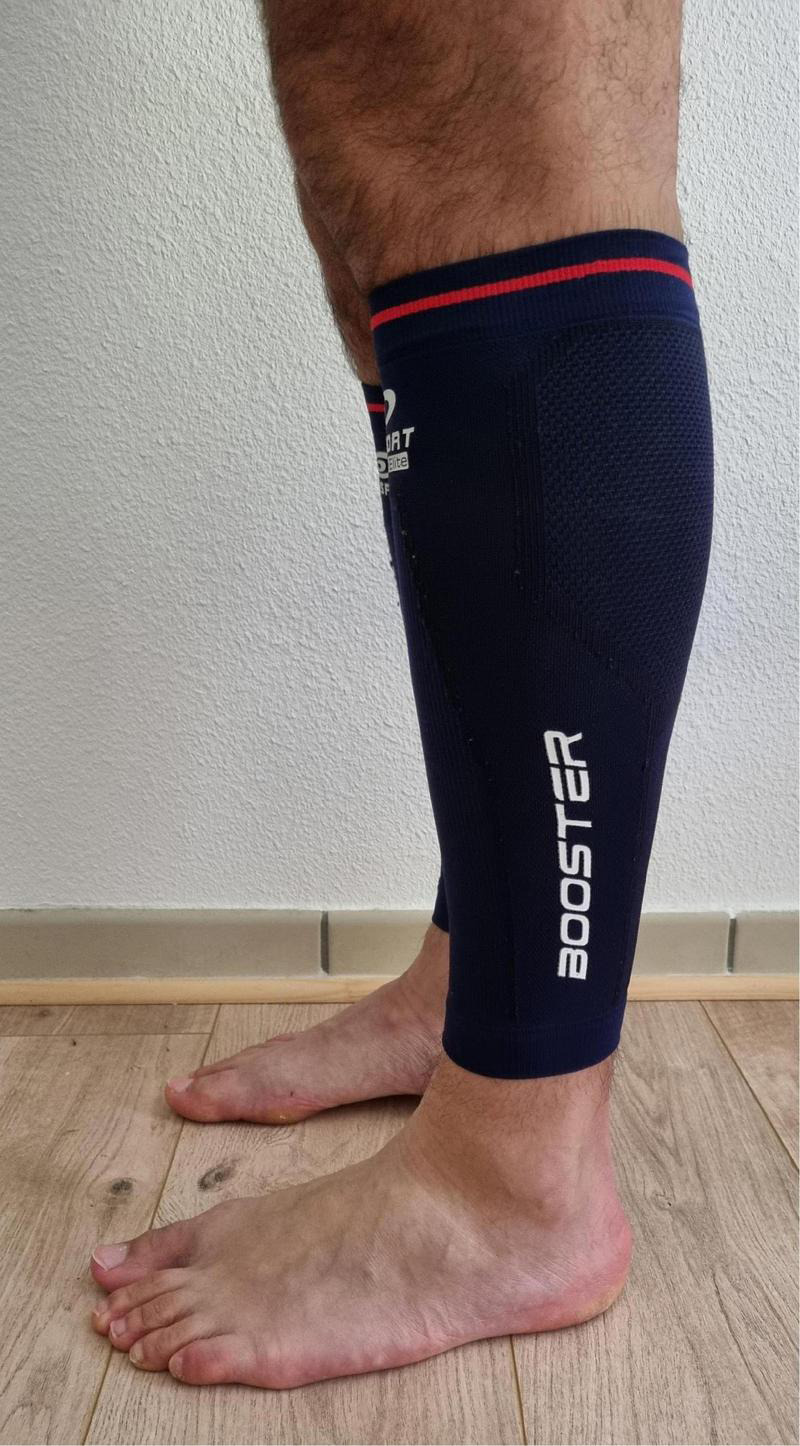
Compression sleeves worn by participants in the compression garments (CG) condition.

Postural tasks were performed with or without wearing compression garments (CG and REF condition, respectively). In the CG condition, calf compression sleeves (Booster, BV sport^®^, Saint Etienne, France) made of 79% Polyamide and 21% Elastane, were worn by the participants (Figure 1). According to the manufacturer’s specifications, these garments provide a pressure that increases gradually from 13 mmHg at the lower part of the calf to 20–25 mmHg at the gastrocnemius belly (20 mmHg at the lateral part and 25 mmHg at the medial part). The size of the compression sleeves was individually fitted according to guidelines of the manufacturers, based on participants’ height and calf circumference. Four sizes were used: M+, L+, XL+, and XXL+, sized for 34–38/>175, 38–43/>175, 38–43/>192, 43–48/192 (calf circumference/height, in cm), respectively, so as to accommodate all participants’ body shape. Participants performed a set of three trials in each postural task (stable and unstable) and condition (REF and CG) with each set completed in a randomized order to avoid any learning effect. The first two trials were not recorded and served as familiarization trials. Since a stable and reliable balance score is achieved at the third trial in a one one-leg stance (Cug and Wikstrom, 2014), only the third trial was recorded and considered for statistical analysis for each postural task and in each experimental condition. Subjects were sitting quietly on a chair for 2 min between balance assessments.

### Analysis of Data

The following parameters of the center of pressure were initially calculated: mean velocity (sum of the cumulated COP displacement divided by the trial time) along the medio-lateral (VX) and antero-posterior (VY) axes and surface area (S: 90% confidence ellipse) (Paillard and Noé, 2015). Based on the method proposed by Read et al. (2018) and Fort-Vanmeerhaeghe et al. (2020), an asymmetry index (ASY) between both legs was then calculated for each center of pressure parameter (ASY_VX, ASY_VY, ASY_S) as follows:


$$\text{ASY}\left(\%\right)=\mathrm{ABS}\left(\frac{\left(\mathrm{Highest}\,\mathrm{performing}\,\mathrm{limb}-\mathrm{Lowest}\,\mathrm{performing}\,\mathrm{limb}\right)}{\mathrm{Highest}\,\mathrm{performing}\,\mathrm{limb}}\right)\times 100,$$

with ABS: absolute value.

When considering that with participants who were asked to sway as little as possible when standing upright, the lower the center of pressure parameters (i.e., VX, VY, S), the more efficient the balance control (Paillard and Noé, 2015), the highest performing limb was then defined as the side with the lowest center of pressure value.

### Statistical Analysis

The two postural tasks (stable and unstable) were analyzed independently. Normality was tested using the Shapiro–Wilk test. As the dependent variables (ASY_VX, ASY_VY, and ASY_S) did not meet the assumption of normal distribution, non-parametric Wilcoxon sign rank tests were applied to determine differences between the CG and REF conditions. The difference of each asymmetry index between the CG and REF conditions (difference = CG - REF) was also calculated to easily differentiate participants who benefit from CG wearing to reduce inter-limb balance asymmetries (negative difference) and those who do not (positive difference). Then, Spearman’s rank order correlations were undertaken between asymmetry indexes at baseline (i.e., in the REF condition) and differences in asymmetry between the REF and CG conditions to assess whether the beneficial effects of CG in reducing inter-limb balance asymmetries was related to the participant’s inter-limb balance asymmetries at baseline.

A qualitative analysis was also carried out to check for a specific influence of compression garments on balance control of the dominant and non-dominant leg, determined by the preferred kicking leg (Paillard and Noé, 2020). A dichotomization method was performed on the center of pressure parameters to characterize three levels of CG influence on balance control: a negative influence, a negligible influence and a positive influence. The following are the thresholds used in the dichotomization procedure: a negative influence was considered when the value in the CG condition exceeded that in the REF condition by more than 10%; a negligible influence was considered when the difference between either conditions did not exceed 10%; a positive influence was considered when the value in the REF condition exceeded that in the CG condition by more than 10%. χ^2^-conformity tests were independently performed on DL and NDL data to test the null hypothesis (similar distribution of individuals for whom CG have a negative, negligible, or positive influence on balance control). This qualitative analysis was performed on the whole sample and on subgroups of individuals in order to check whether individuals who benefited from CG to decrease inter-limb balance asymmetries and those who do not were differently affected by the wearing of CG on the dominant and non-dominant leg. Sub-groups were formed by distinguishing subjects in whom CG wearing had no substantial influence on asymmetry indexes (“unchanged asymmetry with CG” subgroup, i.e., with an increase/decrease of the asymmetry index between REF and CG conditions of less than 10%), subjects in whom CG wearing markedly increased the level of asymmetry (“increase in asymmetry with CG” subgroup, i.e., with an increase of the asymmetry index of more than 10% in the CG condition) and subjects in whom CG wearing markedly decreased the level of asymmetry (“decrease in asymmetry with CG” subgroup, i.e., with a decrease of the asymmetry index of more than 10% in the CG condition). Based on previous studies about reliability of center of pressure-based postural sway measures during one-leg stance in young healthy participants, the 10% threshold was chosen to distinguish between clear and unclear changes due to measurement noise (Lin et al., 2008; Muehlbauer et al., 2011; da Silva et al., 2013). The relationships between subgroups of individuals and specific influence of CG on the dominant and non-dominant leg were then tested with the Fisher’s exact test after having constructed contingency tables. Statistical analyses were performed with R statistical software. The significance level was set at *p* < 0.05.

## Results

**FIGURE 2:**
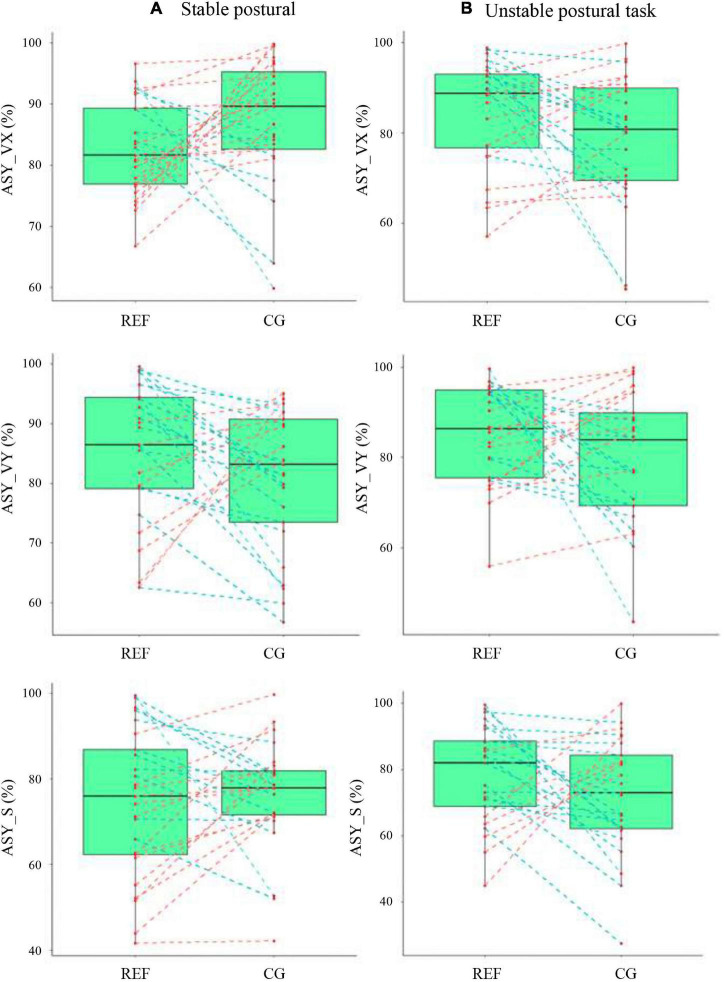
Boxplot representation with individual data points of center of pressure asymmetry indexes in the reference (REF) and compression garments (CG) conditions in **(A)** the stable postural task and **(B)** the unstable postural task. The red dotted lines illustrate individuals whose asymmetry indices increase with CG while the blue dotted lines show individuals whose asymmetry indices decrease with CG.

Figure 2 illustrates boxplots with individual data points of asymmetry indexes in the REF and CG conditions. No significant differences were observed between REF and CG conditions in both the stable and unstable postural tasks. When focusing on the individual data points, one could notice that the asymmetry indexes did not evolve similarly among participants between the REF and CG conditions: asymmetry indexes increased with CG in some participants (red dotted lines on Figure 2), while asymmetry indexes decreased with CG in others (blue dotted lines on Figure 2).

**FIGURE 3:**
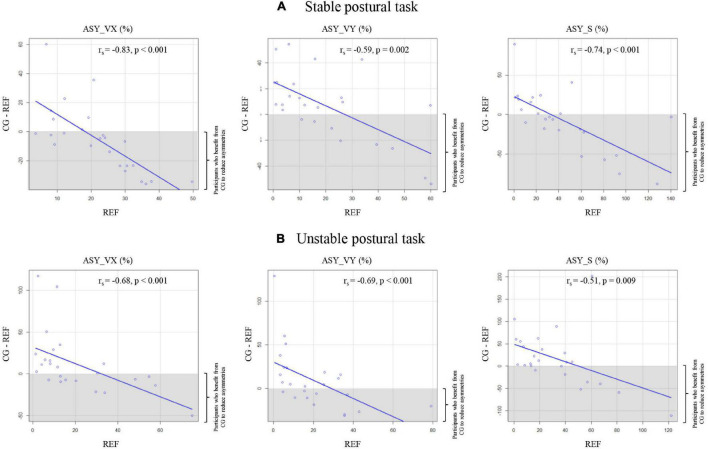
Scatterplots representing the correlations between asymmetry indexes (ASY_VX, ASY_VY, and ASY_S) in the REF condition and the differences in asymmetry between REF and compression garments (CG) conditions (CG–REF) in **(A)** the stable postural task and **(B)** the unstable postural task. Gray areas correspond to participants who benefit from CG wearing to reduce inter-limb balance asymmetries (negative difference). Blank areas correspond to participants who do not benefit from CG wearing to reduce inter-limb balance asymmetries (positive difference). r_*s*_: Spearman’s rank correlation coefficient.

Scatterplots illustrating correlations between ASY indexes in the REF condition and the differences between the CG and REF conditions are shown in Figure 3. In both stable and unstable postural tasks and for all asymmetry indexes, there were significant and large relationships between asymmetry indexes in the REF condition and asymmetry differences between CG and REF conditions. This result indicates that high levels of inter-limb balance asymmetry at baseline [approximate (40–120%) range of asymmetry] was associated with negative differences between CG and REF conditions (i.e., a beneficial effect of CG wearing in reducing inter-limb balance asymmetries). On the contrary, low levels of inter-limb balance asymmetry at baseline [approximate (0–40%) range of asymmetry] were rather associated with positive differences between CG and REF conditions (i.e., an increase of inter-limb balance asymmetries when wearing CG).

Table 1 shows the distribution of participants according to the influence of CG wearing (negative, negligible, or positive influence) on balance control of the dominant and non-dominant leg in the whole sample and in subgroups of individuals. In the stable postural task, results from χ^2^ conformity tests performed in the whole sample were not significant, thus illustrating a homogeneous distribution among participants with a negative, negligible or positive influence of CG wearing on balance control of the dominant or the non-dominant leg. In this postural task, results from the Fisher’s exact test were also not significant. In the unstable postural task, results from the χ^2^ conformity test applied on participants’ distribution based on S data on the dominant-leg showed a significant difference from an equal distribution (χ^2^ = 6.08; *p* < 0.05), with a an under-representation of individuals for whom CG wearing had negligible influence on the dominant-leg. When the χ^2^ test was applied on participants’ distribution based on VY data on the non-dominant leg, a significant difference from an equal distribution (χ^2^ = 11.12; *p* < 0.004), with an over-representation of individuals for whom CG wearing had negligible influence on balance control of the non-dominant leg. Results from the Fisher’s exact test showed a significant association between subgroups of participants and CG influence on balance control on the dominant leg when participants’ distribution were based on VX (*p* < 0.003) and VY data (*p* < 0.005). Individuals in whom CG wearing markedly increased the level of asymmetry were negatively influenced by CG wearing on the dominant leg. Results from the Fisher’s exact test did not show any significant association between subgroups of participants and CG influence on balance control of the non-dominant leg.

**TABLE 1:** Distribution of participants according to the influence of compression garments (CG) wearing (negative, negligible, or positive) on the dominant and non-dominant leg in the whole sample and in subgroups of individuals.

				Stable task			Unstable task		
				Negative	Negligible	Positive	Negative	Negligible	Positive
VX		Whole sample	Dominant leg	9	10	6	10	9	6
			Non-dominant leg	5	12	8	8	11	6
	Subgroups	Decrease in asymmetry with CG	Dominant leg	5	2	3	4	0	3
			Non-dominant leg	2	4	4	1	3	3
		Unchanged asymmetry with CG	Dominant leg	1	6	2	0	6	0
			Non-dominant leg	1	6	2	1	5	0
		Increase in asymmetry with CG	Dominant leg	3	2	1	6	3	3
			Non-dominant leg	2	2	2	6	3	3
VY		Whole sample	Dominant leg	10	8	7	10	7	8
			Non-dominant leg	6	10	9	3	16	6
	Subgroups	Decrease in asymmetry with CG	Dominant leg	3	2	3	3	3	3
			Non-dominant leg	1	4	3	2	5	2
		Unchanged asymmetry with CG	Dominant leg	1	1	0	0	4	0
			Non-dominant leg	1	1	0	0	4	0
		Increase in asymmetry with CG	Dominant leg	6	5	4	7	0	5
			Non-dominant leg	4	5	6	1	7	4
S		Whole sample	Dominant leg	11	3	11	13	3	9
			Non-dominant leg	9	7	9	8	8	9
	Subgroups	Decrease in asymmetry with CG	Dominant leg	3	2	7	4	0	3
			Non-dominant leg	6	3	3	2	3	2
		Unchanged asymmetry with CG	Dominant leg	1	1	2	1	2	1
			Non-dominant leg	0	1	3	0	3	1
		Increase in asymmetry with CG	Dominant leg	7	0	2	8	1	5
			Non-dominant leg	3	3	3	6	2	6

*Subgroups of individuals were formed by distinguishing subjects in whom CG wearing had no substantial influence on asymmetry indexes (“unchanged asymmetry with CG” subgroup), subjects in whom CG wearing markedly increased the level of asymmetry (“increase in asymmetry with CG” subgroup) and subjects in whom CG wearing markedly decreased the level of asymmetry (“decrease in asymmetry with CG” subgroup).*

*VX, mean velocity of the center of pressure along the medio-lateral axis; VY, mean velocity of the center of pressure along the antero-posterior axis; S, center of pressure surface area (90% confidence ellipse).*

## Discussion

The aim of this study was to analyze the effects of CG wearing on inter-limb balance asymmetries. The hypothesis was that CG would reduce inter-limb balance asymmetries, especially in subjects with high level of asymmetries. When using a standard statistical approach with pairwise comparisons between group levels, our results showed that the wearing of CG was not associated with a significant reduction of inter-limb balance asymmetries. However, when analyzing the effect of CG wearing according to participants’ initial level of asymmetry, results from correlation analyses indicated that beneficial effects of CG in reducing inter-limb balance asymmetries were related to participant’s asymmetry levels at baseline. Only participants with high levels of inter-limb balance asymmetries at baseline benefited from CG to decrease inter-limb balance asymmetries.

Inter-limb balance asymmetries are mainly related to differences in somatosensory processing between both legs originating from cerebral hemispheric asymmetry (Promsri et al., 2018, 2019; Marcori et al., 2020; Liu et al., 2021). Within the perspective of the dynamic dominance model of laterality (Sainburg, 2005, 2014), one hemisphere (the left hemisphere for right-handed individuals) would rather be specialized in limb trajectory control, whereas the other hemisphere would rather be specialized for impedance control—i.e., control of limb position and maintenance of a stable posture (Sainburg, 2005, 2014; Marcori et al., 2020). Lateralization of impedance control is based on hemisphere specialization for the utilization of somatosensory cues (Goble et al., 2006; Sainburg, 2014; Liu et al., 2021), which is characterized by the superiority of one hemisphere in processing somatosensory information (the right hemisphere for right-handed individuals), thus resulting in better proprioception of one limb (the left limb for right-handed individuals) compared to the other (Carnahan and Elliott, 1987; Goble et al., 2006). Participants’ injury history and/or experience in asymmetric motor practices can modulate lateralization of impedance control and increase the difference in somatosensory processing between the two legs, thus inducing large inter-individual differences of balance inter-limb asymmetry (Marcori et al., 2019; Paillard and Noé, 2020).

When wearing CG, frictional forces that activate both slow and fast-adapting cutaneous mechanoreceptors are applied to the skin, which can improve joint position sense and balance control (Kuster et al., 1999; You et al., 2004; Cameron et al., 2008; Michael et al., 2014; Woo et al., 2017; Baige et al., 2020; Broatch et al., 2021). In the present study, there was a great heterogeneity in the ability of participants to benefit from CG wearing to reduce inter-limb balance asymmetries. Previous studies about the effects of CG on functional somatosensory abilities have produced concordant findings while reporting a strong inter-individual variability in responses to the wearing of CG in young healthy participants (You et al., 2004; Cameron et al., 2008; Broatch et al., 2021). After ranking participants according to their score in a movement discrimination task, these studies showed that only participants with poor lower limb somatosensation benefited from the wearing of CG to improve joint position sense, thus illustrating that the magnitude of the beneficial effects of CG wearing was inversely related to the participant’s somatosensation at baseline. Similar findings were observed with other stimulation strategies such as ankle taping (Long et al., 2017), textured insoles (Steinberg et al., 2016), and vibration stimulation (Liu et al., 2021).

In the present study, participants with high levels of inter-limb balance asymmetries at baseline benefited from CG wearing to reduce inter-limb balance asymmetries. However, in both the stable and unstable postural tasks, the data from the qualitative analysis about the specific influence of CG on both legs (Table 1) did not illustrate a stronger influence of CG on the dominant leg or the non-dominant leg in individuals who benefited from CG wearing to markedly decrease inter-limb asymmetries. In participants with low levels of inter-limb balance asymmetries at baseline, CG were unhelpful and could also increase inter-limb-balance asymmetries in some participants. Further studies reported analogous findings while showing that proprioceptive acuity decreased in participants with higher levels of proprioceptive ability when they wore external devices that stimulate cutaneous receptors (Cameron et al., 2008; Steinberg et al., 2016; Long et al., 2017; Liu et al., 2021). These authors postulated that in subjects with a high proprioceptive acuity at baseline, the application of an external stimulation device confused and overloaded the sensorimotor system by delivering redundant information, thus resulting in an impaired proprioceptive ability. Liu et al. (2021) noticed that proprioception deteriorated only in the dominant leg in individuals with high proprioceptive acuity following stimulation of the calf muscles with a vibrating foam roller. Our results from the qualitative analysis also suggest that, in the most challenging postural task (i.e., the unstable postural task) CG wearing would have a greater impact on the dominant leg and, more specifically, that CG negatively influenced balance control only on the dominant leg in participants in whom CG wearing increased inter-limb asymmetries. Our results therefore seem to confirm the hypothesis formulated by Liu et al. (2021) about different effects of the application of external stimuli on different hemispheres and show that the non-dominant hemisphere would to be less sensitive to somatosensory stimulation.

A limitation of the current research is the absence of measure of the pressure exerted by the calf compression sleeves. Although compression sleeves were individually fitted by following the manufacturer’s specifications and choosing proper sizing according to individuals’ calf circumference, commercially manufactured compression garments do not provide exactly the same level of compression in all subjects. Potential differences in the pressures exerted by the CG might modulate the effects of CG on inter-limb balance asymmetries. Nevertheless, studies that have tested the effects of CG providing various compression levels on balance control did not report any significant differences attributed to the level of compression (e.g., Hijmans et al., 2009; Jaakkola et al., 2017; Woo et al., 2018). It should be noted that the compression sleeves used in this study did not cover the ankle joint, whereas somatosensory information from the ankle joint plays an essential role in balance control (Han et al., 2015). Hence, one can hypothesize that the use of CG covering the ankle joints such as compression socks could potentially provide larger benefits in reducing inter-limb balance asymmetries. Further experiments are needed to explore the influence of various designs of CG (e.g., full leg CG, compression socks, calf and knee compression sleeves) on inter-limb balance asymmetries.

The present study provides new insights about the influence of CG on inter-limb balance asymmetries. From a practical point of view, these results suggest that CG wearing could reduce inter-limb balance asymmetries and the related injury risk in participants with high levels of inter-limb balance asymmetries at baseline. In return, since it rather increases asymmetries in participants having low baseline balance asymmetries, CG wearing should be avoided in these participants.

## Data Availability Statement

The raw data supporting the conclusions of this article will be made available by the authors, without undue reservation.

## Ethics Statement

The studies involving human participants were reviewed and approved by the University of Pau and Adour Countries Ethics Committee. The patients/participants provided their written informed consent to participate in this study.

## Author Contributions

FN, KB, and TP designed the study. KB acquired the data. FN analyzed the data and wrote the manuscript, while KB and TP revised it. All authors signed the final approval for publication.

## Conflict of Interest

The authors declare that the research was conducted in the absence of any commercial or financial relationships that could be construed as a potential conflict of interest.

## Publisher’s Note

All claims expressed in this article are solely those of the authors and do not necessarily represent those of their affiliated organizations, or those of the publisher, the editors and the reviewers. Any product that may be evaluated in this article, or claim that may be made by its manufacturer, is not guaranteed or endorsed by the publisher.

## References

[B1] Auerbach B. M. Ruff C. B. (2006). Limb bone bilateral asymmetry: variability and commonality among modern humans. *J. Hum. Evol.* 50 203–218. 10.1016/j.jhevol.2005.09.004 16310833

[B2] Baige K. Noé F. Bru N. Paillard T. (2020). Effects of compression garments on balance control in young healthy active subjects: a hierarchical cluster analysis. *Front. Hum. Neurosci.* 14:582514. 10.3389/fnhum.2020.582514 33281583 PMC7689056

[B3] Barrera-Domínguez F. J. Carmona-Gómez A. Tornero-Quiñones I. Sáez-Padilla J. Sierra-Robles Á Molina-López J. (2021). Influence of dynamic balance on jumping-based asymmetries in team sport: a between-sports comparison in basketball and handball athletes. *Int. J. Environ. Res. Public Health* 18:1866. 10.3390/ijerph18041866 33672951 PMC7917681

[B4] Bishop C. Turner A. Read P. (2018). Effects of inter-limb asymmetries on physical and sports performance?: a systematic review. *J. Sports Sci.* 36 1135–1144. 10.1080/02640414.2017.1361894 28767317

[B5] Broatch J. R. Halson S. L. Panchuk D. Bishop D. J. Waddington G. (2021). Compression enhances lower-limb somatosensation in individuals with poor somatosensation, but impairs performance in individuals wth good somatosensation. *Transl. Sports Med.* 4 280–288. 10.1002/tsm2.214

[B6] Cameron M. L. Adams R. D. Maher C. G. (2008). The effect of neoprene shorts on leg proprioception in Australian football players. *J. Sci. Med. Sport.* 11 345–352. 10.1016/j.jsams.2007.03.007 17889610

[B7] Carnahan H. Elliott D. (1987). Pedal asymmetry in the reproduction of spatial locations. *Cortex* 23 157–159. 10.1016/s0010-9452(87)80028-x 3568704

[B8] Chisholm A. E. Perry S. D. McIlroy W. E. (2011). Inter-limb centre of pressure symmetry during gait among stroke survivors. *Gait Posture* 33 238–243. 10.1016/j.gaitpost.2010.11.012 21167716

[B9] Cug M. Wikstrom E. A. (2014). Learning effects associated with the least stable level of the Biodex§stability system during dual and single limb stance. *J. Sports Sci. Med.* 60 22–26. 10.5152/tftrd.2014.59354 PMC3990894 24790494

[B10] da Silva R. A. Bilodeau M. Parreira R. B. Teixeira D. C. Amorim C. F. (2013). Age-related differences in time-limit performance and force platform-based balance measures during one-leg stance. *J. Electromyogr. Kinesiol.* 23 634–639. 10.1016/j.jelekin.2013.01.008 23403137

[B11] Dos’Santos T. Bishop C. Thomas C. Comfort P. Jones P. A. (2019). The effect of limb dominance on change of direction biomechanics: a systematic review of its importance for injury risk. *Phys. Ther. Sport.* 37 179–189. 10.1016/j.ptsp.2019.04.005 30986764

[B12] Fort-Vanmeerhaeghe A. Bishop C. Buscà B. Aguilera-Castells J. Vicens-Bordas J. Gonzalo-Skok O. (2020). Inter-limb asymmetries are associated with decrements in physical performance in youth elite team sports athletes. *PLoS One* 15:e0229440. 10.1371/journal.pone.0229440 32126107 PMC7053777

[B13] Ghai S. Driller M. W. Masters R. S. W. (2018). The influence of below-knee compression garments on knee-joint proprioception. *Gait Posture* 60 258–261. 10.1016/j.gaitpost.2016.08.008 27523397

[B14] Goble D. J. Lewis C. A. Brown S. H. (2006). Upper limb asymmetries in the utilization of proprioceptive feedback. *Exp. Brain Res.* 168 307–311. 10.1007/s00221-005-0280-y 16311728

[B15] Han J. Anson J. Waddington G. Adams R. Liu Y. (2015). The role of ankle proprioception for balance control in relation to sports performance and injury. *Biomed. Res. Int.* 2015:842804. 10.1155/2015/842804 26583139 PMC4637080

[B16] Hasan H. Davids K. Chow J. Y. Kerr G. (2016). Compression and texture in socks enhance football kicking performance. *Hum. Mov. Sci.* 48 102–111. 10.1016/j.humov.2016.04.008 27155962

[B17] Hijmans J. M. Zijlstra W. Geertzen J. H. B. Hof A. L. Postema K. (2009). Foot and ankle compression improves joint position sense but not bipedal stance in older people. *Gait Posture* 29 322–325. 10.1016/j.gaitpost.2008.10.051 19019679

[B18] Jaakkola T. Linnamo V. Woo M. T. Davids K. Piirainen J. M. Gråstén A. (2017). Effects of training on postural control and agility when wearing socks of different compression levels. *Biomed. Hum. Kin.* 9 107–114. 10.1515/bhk-2017-0016

[B19] Kuster M. S. Grob K. Kuster M. Wood G. A. Chter A. (1999). The benefits of wearing a compression sleeve after ACL reconstruction. *Med. Sci. Sports Exerc.* 31 368–371. 10.1097/00005768-199903000-00003 10188739

[B20] Lin D. Seol H. Nussbaum M. A. Madigan M. L. (2008). Reliability of COP-based postural sway measures and age-related differences. *Gait Posture* 28 337–342. 10.1016/j.gaitpost.2008.01.005 18316191

[B21] Liu B. Witchalls J. Waddington G. Adams R. Wu S. Han J. (2021). Vibration of calf muscles has reverse effects on right and left ankle proprioception in high and low proprioceptive performer groups, Somatosens. *Mot. Res.* 38 101–107. 10.1080/08990220.2020.1860929 33345696

[B22] Long Z. Wang R. Han J. Waddington G. Adams R. Anson J. (2017). Optimizing ankle performance when taped: effects of kinesiology and athletic taping on proprioception in full weight-bearing stance. *J. Sci. Med. Sport.* 20 236–240. 10.1016/j.jsams.2016.08.024 27686616

[B23] Madruga-Parera M. Bishop C. Fort-Vanmeerhaeghe A. Beato M. Gonzalo-Skok O. Romero-Rodríguez D. (2020). Effects of 8 weeks of isoinertial vs. cable-resistance training on motor skills Performance and interlimb asymmetries. *J. Strength Cond. Res.* [Epub online ahead of print]. 10.1519/jsc.0000000000003594 32379241

[B24] Marcori A. J. Monteiro P. H. M. Okazaki V. H. A. (2019). Changing handedness: what can we learn from preference shift studies? *Neurosci. Biobehav. Rev.* 107 313–319. 10.1016/j.neubiorev.2019.09.019 31521700

[B25] Marcori A. J. Teixeira L. A. Dascal J. B. Okazaki V. H. A. (2020). Are the predictions of the dynamic dominance model of laterality applicable to the lower limbs? *Hum. Mov. Sci.* 73:102684. 10.1016/j.humov.2020.102684 32950842

[B26] Michael J. S. Dogramaci S. N. Steel K. A. Graham K. S. (2014). What is the effect of compression garments on a balance task in female athletes? *Gait Posture* 39 804–809. 10.1016/j.gaitpost.2013.11.001 24314813

[B27] Mohammadi F. Salavati M. Akhbari B. Mazaheri M. Khorrami M. Negahban H. (2012). Static and dynamic postural control in competitive athletes after anterior cruciate ligament reconstruction and controls. *Knee Surg. Sports Traumatol. Arthrosc.* 20 1603–1610. 10.1007/s00167-011-1806-4 22124847

[B28] Muehlbauer T. Roth R. Mueller S. Granacher U. (2011). Intra and intersession reliability of balance measures during one-leg standing in young adults. *J. Strength Cond. Res.* 25 2228–2234. 10.1519/JSC.0b013e3181fb393b 21685809

[B29] Paillard T. (2017). Plasticity of the postural function to sport and/or motor experience. *Neurosci. Biobehav. Rev.* 72 129–152. 10.1016/j.neubiorev.2016.11.015 27894829

[B30] Paillard T. Noé F. (2015). Techniques and methods for testing the postural function in healthy and pathological subjects. *Biomed. Res. Int.* 2015:891390. 10.1155/2015/891390 26640800 PMC4659957

[B31] Paillard T. Noé F. (2020). Does monopedal postural balance differ between the dominant leg and the non-dominant leg? A review. *Hum. Mov. Sci.* 74:102686. 10.1016/j.humov.2020.102686 33059226

[B32] Pardos-Mainer E. Casajús J. A. Gonzalo-Skok O. (2019). Adolescent female soccer players’ soccer-specific warm-up effects on performance and inter-limb asymmetries. *Biol. Sport.* 36 199–207. 10.5114/biolsport.2019.85453 31624413 PMC6786331

[B33] Pau M. Leban B. Deidda M. Porta M. Coghe G. Cattaneo D. (2021). Use of wrist-worn accelerometers to quantify bilateral upper limb activity and asymmetry under free-living conditions in people with multiple sclerosis. *Mult. Scler. Relat. Disord.* 53:103081. 10.1016/j.msard.2021.103081 34166981

[B34] Promsri A. Haid T. Federolf P. (2018). How does lower limb dominance influence postural control movements during single leg stance? *Hum. Mov. Sci.* 58 165–174. 10.1016/j.humov.2018.02.003 29448161

[B35] Promsri A. Longo A. Haid T. Doix A. M. Federolf P. (2019). Leg dominance as a risk factor for lower-limb injuries in downhill skiers-a pilot study into possible mechanisms. *Int. J. Environ. Res. Public Health* 16:3399. 10.3390/ijerph16183399 31540226 PMC6765833

[B36] Read P. J. Oliver J. L. Myer G. D. De Ste Croix M. B. A. Lloyd R. S. (2018). The effects of maturation on measures of asymmetry during neuromuscular control tests in elite male youth soccer players. *Pediatr. Exerc. Sci.* 30 168–175. 10.1123/pes.2017-0081 28787266 PMC6538932

[B37] Sainburg R. L. (2005). Handedness: differential specializations for control of trajectory and position. *Exerc. Sport Sci. Rev.* 33 206–213. 10.1097/00003677-200510000-00010 16239839 PMC10709818

[B38] Sainburg R. L. (2014). Convergent models of handedness and brain lateralization. *Front. Psychol.* 5:1092. 10.3389/fpsyg.2014.01092 25339923 PMC4189332

[B39] Schneiders A. G. Sullivan S. J. O’Malley K. J. Clarke S. V. Knappstein S. A. Taylor L. J. (2010). A valid and reliable clinical determination of footedness. *PM R* 2 835–841. 10.1016/j.pmrj.2010.06.004 20869683

[B40] Steib S. Zahn P. Zu Eulenburg C. Pfeifer K. Zech A. (2016). Time-dependent postural control adaptations following a neuromuscular warm-up in female handball players: a randomized controlled trial. *BMC Sports Sci. Med. Rehabil.* 8:33. 10.1186/s13102-016-0058-5 27757240 PMC5064777

[B41] Steinberg N. Waddington G. Adams R. Karin J. Tirosh O. (2016). The effect of textured ballet shoe insoles on ankle proprioception in dancers. *Phys. Ther. Sport.* 17 38–44. 10.1016/j.ptsp.2015.04.001 26563491

[B42] Stiffler M. R. Bell D. R. Sanfilippo J. L. Hetzel S. J. Pickett K. A. Heiderscheit B. C. (2017). Star excursion balance test anterior asymmetry is associated with injury status in division I collegiate athletes. *J. Orthop. Sports Phys. Ther.* 47 339–346. 10.2519/jospt.2017.6974 28355980

[B43] Woo M. T. Davids K. Liukkonen J. Chow J. Y. Jaakkola T. (2018). Immediate effects of wearing knee length socks differing in compression level on postural regulation in community-dwelling, healthy, elderly men and women. *Gait Posture* 66 63–69. 10.1016/j.gaitpost.2018.08.011 30165286

[B44] Woo M. T. Davids K. Liukkonen J. Orth D. Chow J. Y. Jaakkola T. (2017). Effects of different lower-limb sensory stimulation strategies on postural regulation-A systematic review and meta-analysis. *PLoS One* 29:e0174522. 10.1371/journal.pone.0174522 28355265 PMC5371369

[B45] Yang J. H. Suh S. W. Sung P. S. Park W. H. (2013). Asymmetrical gait in adolescents with idiopathic scoliosis. *Eur. Spine J.* 22 2407–2413. 10.1007/s00586-013-2845-y 23732766 PMC3886502

[B46] You S. H. Granata K. P. Bunker L. K. (2004). Effects of circumferential ankle pressure on ankle proprioception, stiffness, and postural stability: a preliminary investigation. *J. Orthop. Sports Phys. Ther.* 34 449–460. 10.2519/jospt.2004.34.8.449 15373008

